# Monostotic fibrous dysplasia of a lumbar vertebral body with secondary aneurysmal bone cyst formation: a case report

**DOI:** 10.4076/1752-1947-3-7227

**Published:** 2009-06-24

**Authors:** Marieke N Snieders, Folkert J van Kemenade, Barend J van Royen

**Affiliations:** 1 Department of Orthopaedic Surgery, VU University Medical Center, De Boelelaan, Amsterdam, The Netherlands; 2 Department of Pathology, VU University Medical Center, De Boelelaan, Amsterdam, The Netherlands

## Abstract

We report the case of a 25-year-old Caucasian woman with symptomatic monostotic fibrous dysplasia of the fourth lumbar vertebral body. The patient suffered from a five-week history of progressive low back pain, radiating continuously to the left leg. Her medical history and physical and neurological examination did not demonstrate any significant abnormalities. Radiographs, computed tomography and magnetic resonance imaging revealed an osteolytic expansive lesion with a cystic component of the fourth lumbar vertebral body. Percutaneous transpedicular biopsy showed histological characteristics of fibrous dysplasia superimposed by the formation of aneurysmal bone cyst components. The patient was treated by subtotal vertebrectomy of the L4 vertebral body with anterior reconstruction and her postoperative course was uncomplicated. To our knowledge, this is the first reported case of a monostotic fibrous dysplasia with superimposed secondary aneurysmal bone cysts of a lumbar vertebral body.

## Introduction

Fibrous dysplasia (FD) is a rare but well known benign intramedullary fibro-osseous lesion which may involve one or more bones. It is a developmental condition in which areas of the skeleton fail to mature normally, most often presenting in the long bones of the legs, arms, ribs, pelvis and in the craniofacial bones [1,2]. FD can occur monostotically (MFD, single lesion), polyostotically (PFD, multiple lesions), or in association with café-au-lait skin spots and precocious puberty (McCune-Albright syndrome) [2,3]. Histologically, the manifestation of both forms of the disease is similar, characterized by cellular fibrous tissue with immature trabeculae and disordered islands of bone formation with remarkably little osteoblastic rimming.

Reports describing FD lesions of the spine are limited and most commonly involve the vertebral bodies and adjacent pedicles [4]. There seems to be no predilection for any part of the spinal column, however, sacral or coccygeal involvement is extremely rare [4] and few reports on involvement of the lumbar spine have been published [1,4]-[7].

As with many bone abnormalities, FD can be superimposed by the formation of aneurysmal bone cysts (ABC) [8]-[12]. An ABC is a benign cystic lesion of bone, composed of blood filled spaces separated by connective tissue septa with fibroblasts and osteoclast-like giant cells and reactive woven bone. The woven bone in this lesion is rimmed with osteoblasts and usually follows contours of the septa. It may develop de novo or secondary complicating other benign and malignant bone tumours. In approximately 20% to 30% of cases, an ABC is associated with an underlying skeletal lesion, including FD [8,13,14]. These lesions are referred to as secondary ABC.

Spinal localisation of FD with superimposed secondary ABC is extremely rare. We are aware of only one case report describing secondary ABC formation complicating FD of the cervical spine [9]. To the best of our knowledge, a MFD of the lumbar spine having an ABC component has never been reported before. We present a case of a 25-year-old woman treated for a symptomatic MFD of the fourth lumbar vertebral body with an ABC component.

## Case presentation

**Figure 1:**
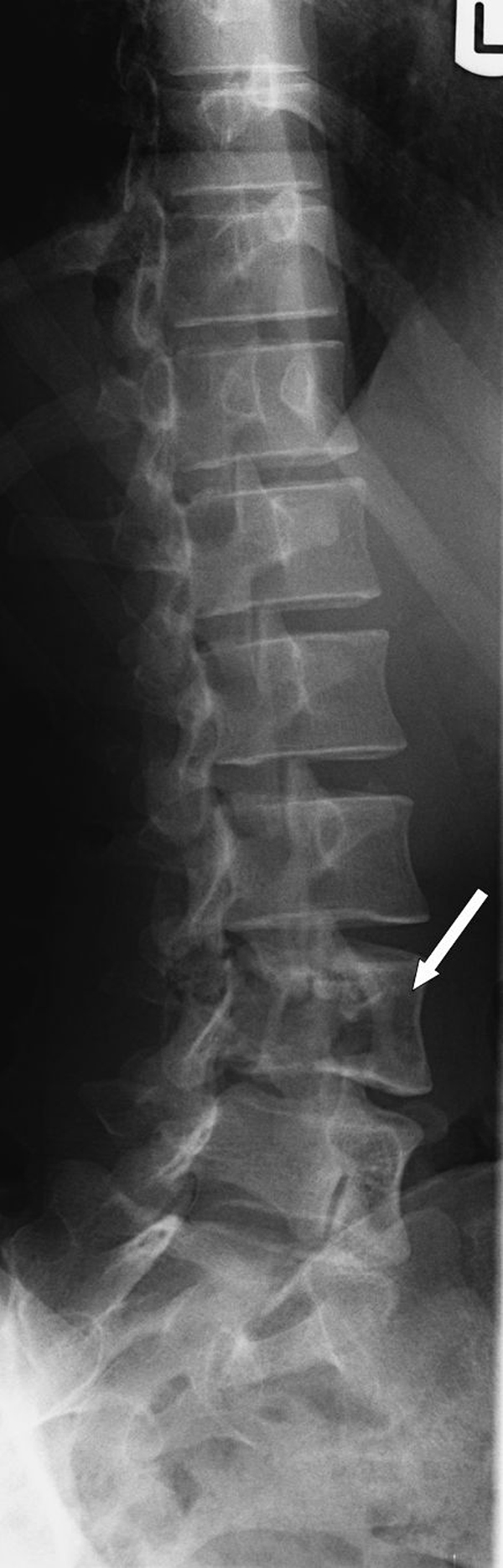
**Left oblique radiograph of the lumbar spine demonstrating radiolucency of vertebra L4**.

**Figure 2:**
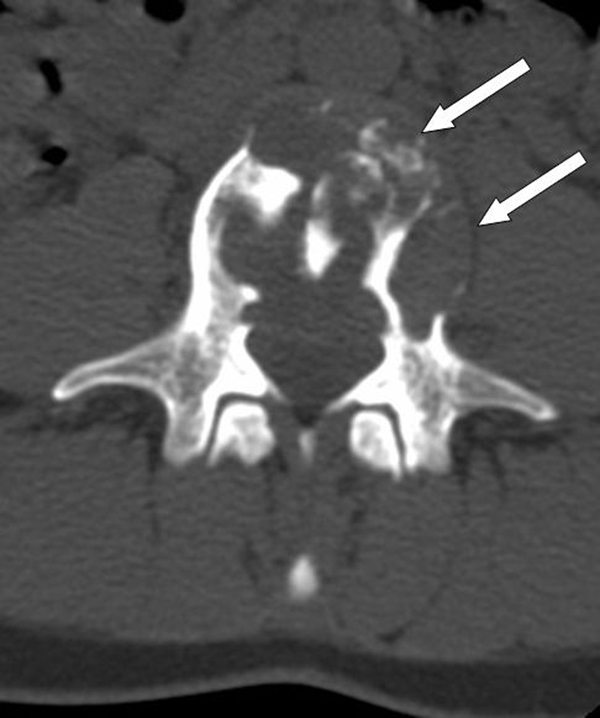
**Transversal CT image of the L4 vertebral body, demonstrating remodelling in partially sclerotic bone**.

**Figure 3:**
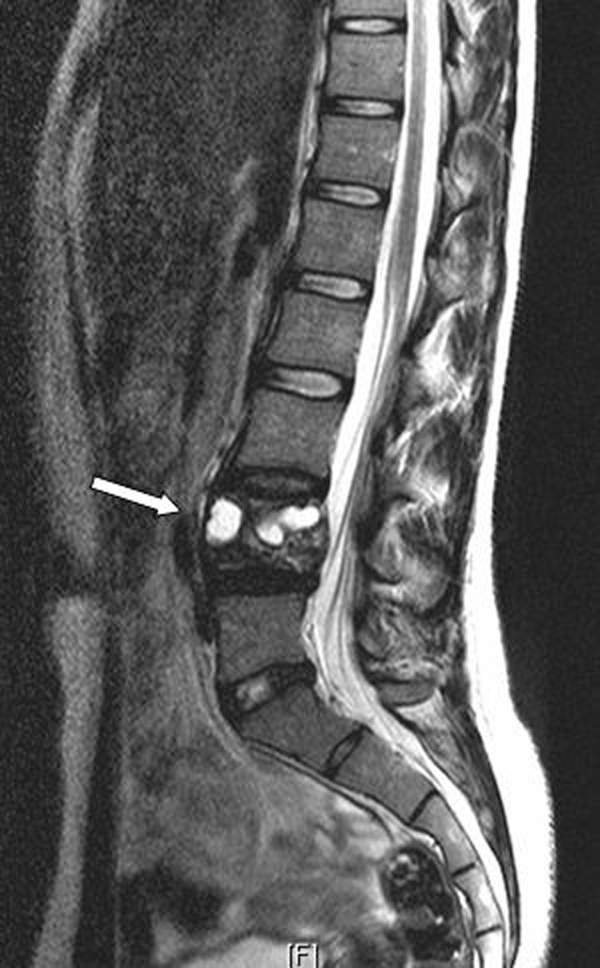
**Sagittal T2-weighted MRI demonstrating cystic components of vertebra L4**.

**Figure 4:**
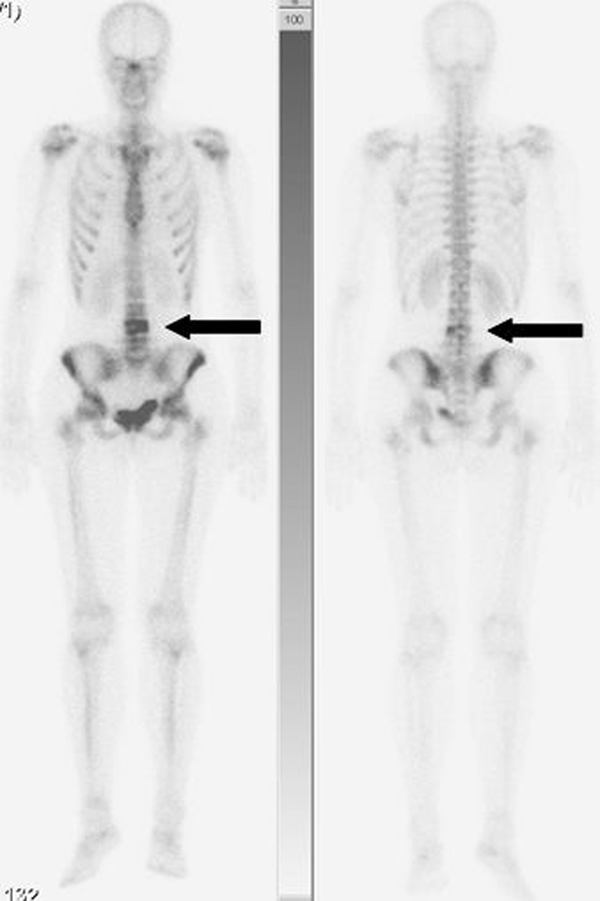
**Whole-body bone scintigraphy with 99mTc-HDP showing an intense, local enhanced uptake at level L4 with areas of decreased uptake demonstrating the cystic components**.

**Figure 5:**
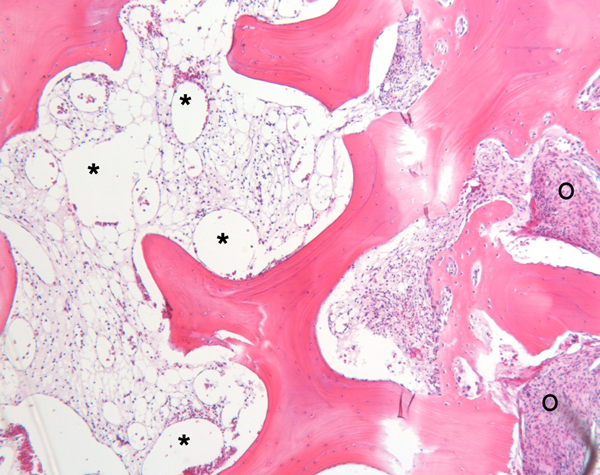
HE-stained (5 x objective) decalcified percutaneous transpedicular biopsy showing on the left side aneurysmatic structures (*), and on the right side margins of fibrous dysplasia (O) infringing on laminar bone structures.

**Figure 6:**
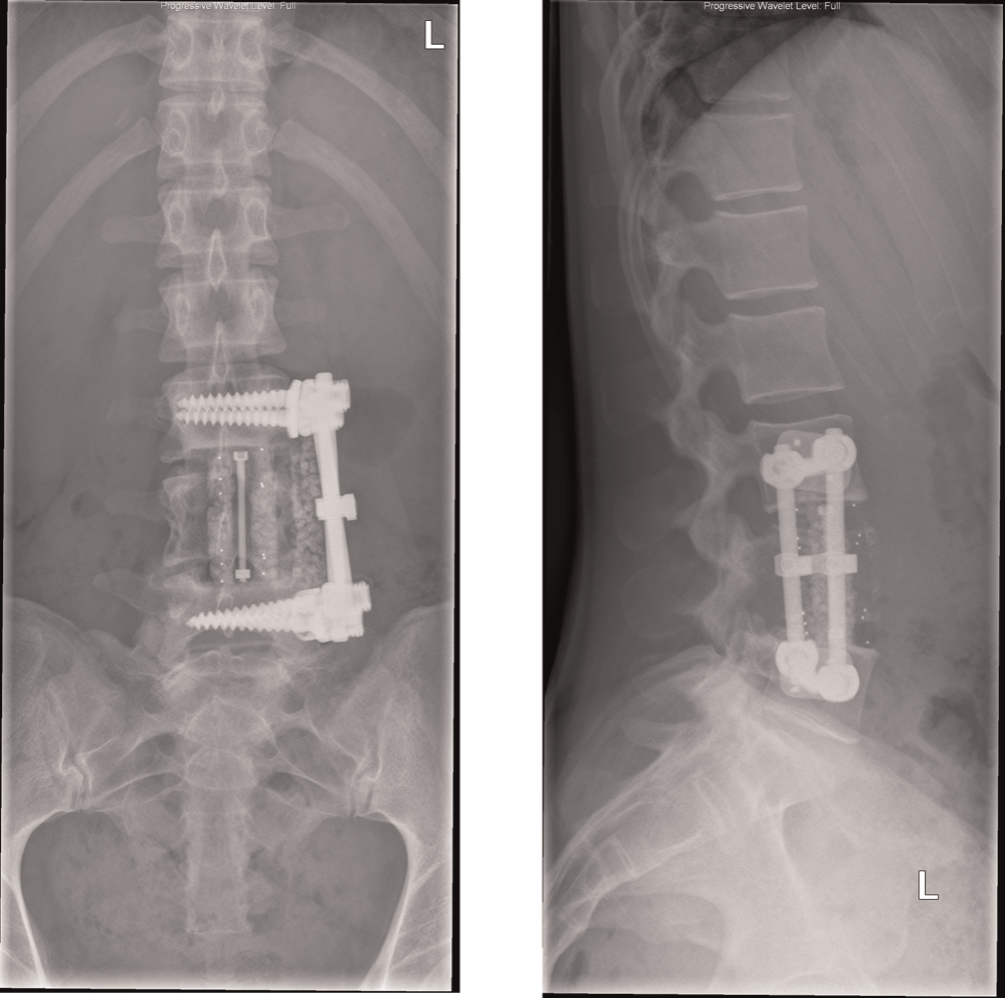
**Postoperative AP and lateral radiograph demonstrating the subtotal vertebrectomy of the L4 vertebral body and anterior reconstruction of the defect with the use of an anterior stackable cage and anterior instrumentation**.

A previously healthy, 25-year-old Caucasian woman presented to our outpatient clinic with a five-week history of progressive low back pain, radiating continuously to the left leg. Her past medical history and her physical and neurological examination revealed no significant abnormalities. Temporary loss of sensibility in the dermatome of L4 was reported, which resolved spontaneously. A blood investigation demonstrated low serum calcium levels of 2.04 mg/dl (2.20-2.60 mg/dl) with normal alkaline phosphate levels of 42 U/L (40-120 U/L). There were no clinical signs of endocrinopathies or café-au-lait hyperpigmentation of the skin. Radiographs of the lumbar spine showed an isolated radiolucency of the fourth lumbar vertebral body (Figure 1). Computed tomography (CT) imaging showed an expansive lesion of the L4 vertebral body with partial bone destruction (Figure 2). The bone of the vertebral body showed signs of partial remodelling and sclerosis and there was cortical destruction of the posterior wall with some epidural extension. Magnetic resonance (MR) imaging following intravenous gadolinium administration displayed a heterogeneous enhancement of the lesion (Figure 3). Cystic components were identified; blood fluid levels within these cysts, however, were absent. Differential diagnosis included an ABC and giant cell tumour. Whole-body bone scintigraphy with 99mTc-HDP (oxidronate) showed an intense, local enhanced uptake at level L4 with areas of decreased uptake demonstrating the cystic components (Figure 4). There were no other locations of 99mTc-HDP enhancement. Percutaneous transpedicular biopsy of the lesion was performed. Histological examination of this biopsy showed characteristic features of both FD and the formation of ABC (Figure 5). The patient underwent subtotal vertebrectomy of the L4 vertebral body including curettage of the entire cyst wall, all abnormal spongy tissues and bone surfaces that were lined with fragile and hypervascular membranes. Stabilization and reconstruction of the defect was performed with the use of an anterior stackable cage filled with tricalcium phosphate granulate and anterior instrumentation. The postoperative course was uncomplicated. At 24 months follow-up radiographs and CT imaging showed a progressive fusion of the anterior fixation with no signs of recurrence (Figure 6).

## Discussion

We describe a patient with a localized symptomatic expansive lesion of the L4 vertebral body with partial bone destruction. Histological examination demonstrated the coexistence of both FD and ABC. FD is a primary benign intramedullary fibro-osseous lesion. However, ABC is a benign vascular bone lesion that may develop de novo or secondary to a pre-existing benign or malignant bone tumour [8,12]-[14]. In our case, the ABC is regarded as a secondary vascular phenomenon superimposed on a pre-existing benign MFD of the fourth lumbar vertebra.

The relation between the ABC and FD of bone is uncommon and has been reported in the literature in only about 35 cases [9,11,12,15]-[17]. Localisation of coexisting FD and secondary ABC has been reported in long bones, ribs, and craniofacial bones. However, to our best knowledge, only one case has been reported with MFD complicated by ABC formation in the cervical spine [9]. This can be explained by the fact that spinal involvement of MFD is very rare, and has been reported in only 25 cases in the literature [4]-[7].

Generally, MFD lesions are asymptomatic and mature after skeletal growth ceases. A MFD lesion is often discovered when radiographs are made for other reasons [2]. The radiographic features of FD are characterized by an expanding greyish 'ground-glass' pattern of cancellous bone with endosteal scalloping cortical bone. In these cases, radionuclide bone scintigraphy is useful to determine whether there is additional skeletal involvement with FD. However, false-negative results can occur in a silent lytic lesion [18]. In addition CT and contrast MRI of the spine are indicated to demonstrate the extend of the lesion [2,19].

The exact pathogenesis of secondary ABC remains unknown. As a result of specific pathophysiological changes, probably resulting from a tumour-induced anomalous vascular process, a secondary reactive non-neoplastic lesion of bone can superimpose the primary lesion [12]. Rapid growth of the secondary ABC with destruction of the intramedullary and juxtacortical structures of the vertebral body, in combination with the dysplastic bone, may lead to pathological stress or fatigue fractures. The diagnosis can be achieved by plain radiographs, MRI and CT. In some cases a fluid-fluid level is clearly visible, but biopsy is mandatory for histological confirmation.

A pathological fracture of the lesion probably causes the initiating symptoms in patients with MFD with superimposed secondary ABC. Our case also did not have any preceding signs of low back pain until five weeks before presentation. We considered an acute bone collapse and soft tissue invasion, in combination with a pathological stress fracture in the dysplastic bone of the vertebra L4, at the beginning of symptomatic deterioration.

The advised treatment for primary ABC of the spine is selective arterial embolization, intralesional excision or surgical en bloc resection [13,19,20]. However, treatment of secondary ABC is defined by the treatment of the pre-existing lesion [8]. Thus, following this paradigm, our patient was treated for MFD of the fourth lumbar vertebral body. MFD lesions are regarded as being not progressive and can be treated by observation and patient education. Malignant transformation of MFD is very rare [2]. However, MFD of the spine has been reported occasionally to continue to grow in skeletally mature patients [4]. Therefore, aggressive en bloc resection and stabilization is the treatment of choice in MFD and secondary ABC with pathological fractures of the spine [13,19,20]. In our case, we performed subtotal vertebrectomy of the L4 vertebral body with curettage of the entire cyst wall. Stabilization and reconstruction of the defect was performed with the use of an anterior stackable cage filled with tricalcium phosphate granulate and anterior instrumentation. At 24 months follow-up, our patient did not have any signs of a local recurrence.

In conclusion, we present the first patient in the literature with a secondary ABC engrafted on an MFD lesion of the fourth lumbar vertebral body. Subtotal vertebrectomy of the L4 vertebral body with curettage of the entire cyst wall and subsequent stabilization and anterior reconstruction of the defect resulted in an excellent outcome.

## Abbreviations

ABC: aneurysmal bone cysts; CT: computed tomography; FD: fibrous dysplasia; MFD: monostotic fibrous dysplasia; MRI: magnetic resonance imaging; PFD: polyostotic fibrous dysplasia.

## Consent

Written informed consent was obtained from the patient for publication of this case report and accompanying images. A copy of the written consent is available for review by the Editor-in-Chief of this journal.

## Competing interests

The authors declare that they have no competing interests.

## Authors' contributions

BJvR and MNS analysed and interpreted the patient data, performed the surgery, and were major contributors in writing the manuscript. FJvK performed the histological examination of the biopsy and final vertebral body. All authors read and approved the final manuscript.
